# Pneumovesicoscopic ureteral reimplantation for pediatric vesicoureteral reflux: Politano-Leadbetter vs. Cohen technique

**DOI:** 10.3389/fped.2026.1855283

**Published:** 2026-07-21

**Authors:** Mingliu Huang, Ting Zhang, Chao Wang, Shu Dai, Hongliang Xia, Xu Cao, Mingcui Fu, Xiangming Yan, Yunli Bi, Yun Zhou

**Affiliations:** Department of Urology, Children's Hospital of Soochow University, Suzhou, China

**Keywords:** Cohen technique, minimally invasive surgery, pneumovesicoscopy, Politano–Leadbetter technique, vesicoureteral reflux

## Abstract

**Purpose:**

To investigate and compare the clinical efficacy of pneumovesicoscopic Politano–Leadbetter and Cohen ureteral reimplantation for primary vesicoureteral reflux (VUR) in children.

**Methods:**

We retrospectively reviewed 179 children with primary VUR (high-risk grade III and grades IV–V) who underwent pneumovesicoscopic ureteral reimplantation at our institution between October 2021 and January 2024. Patients were allocated to the Politano–Leadbetter group (Group P, *n* = 97) or the Cohen group (Group C, *n* = 82), including 120 unilateral and 59 bilateral cases. Perioperative parameters and postoperative complications were compared over a 6–30 month follow-up period.

**Results:**

All procedures were completed pneumovesicoscopically without conversion to open surgery. In unilateral cases, there were no significant between-group differences in operative time (119.85 ± 29.13 vs. 116.21 ± 22.04 min, *p* = 0.437), estimated blood loss (6.15 ± 0.98 vs. 6.02 ± 0.91 mL, *p* = 0.454), duration of urethral catheterization (4.31 ± 0.59 vs. 4.53 ± 0.85 days, *p* = 0.112), length of hospital stay (6.34 ± 1.71 vs. 6.42 ± 0.93 days, *p* = 0.745), or complication rate (7.5% vs. 15.1%, *p* = 0.217). Similar findings were observed for bilateral cases (all *p* > 0.05). Surgical failure occurred in 2 ureters in Group P and 3 ureters in Group C, the imaging-confirmed anatomical success rate was 98.1% in Group P and 96.3% in Group C.

**Conclusion:**

Pneumovesicoscopic ureteral reimplantation is a safe and effective minimally invasive approach for pediatric VUR in the short term. Although the Politano–Leadbetter technique is technically more demanding, it provides a more physiological ureteral course. Long-term outcomes require further validation.

## Introduction

Primary vesicoureteral reflux (VUR) is a common congenital anomaly of the urinary tract in children, with a reported incidence of 1%–2% (1). VUR predisposes affected children to febrile urinary tract infections and may lead to renal scarring and long-term renal impairment (2). Although open ureteral reimplantation remains highly effective and has well-established outcomes (3, 4), surgical management has increasingly shifted toward minimally invasive approaches over the past two decades. Laparoscopic, robotic-assisted, and pneumovesicoscopic ureteral reimplantation have been adopted worldwide, offering efficacy comparable to open surgery while reducing tissue trauma and accelerating recovery (5–9).

Prior studies have primarily compared open repair with individual minimally invasive techniques (10). However, direct comparisons between pneumovesicoscopic intravesical approaches—particularly the Cohen and Politano–Leadbetter techniques—remain limited, especially with respect to anatomical reconstruction and midterm outcomes in children with high-grade reflux.

In this retrospective study, we analyzed 179 pediatric patients with primary VUR (high-risk grade III and grades IV–V) who underwent pneumovesicoscopic ureteral reimplantation. We compared perioperative parameters and postoperative outcomes over a 6–30 month follow-up period between the pneumovesicoscopic Politano–Leadbetter and Cohen techniques. In addition, we describe key anatomical landmarks and operative refinements intended to simplify the technically demanding step of extravesical ureteral identification during the pneumovesicoscopic Politano–Leadbetter procedure.

## Materials and methods

### Study designs

This retrospective study included 179 pediatric patients who underwent pneumovesicoscopic ureteral reimplantation at our institution between October 2021 and January 2024. All patients received preoperative VCUG, DMSA and DTPA scans. The inclusion criteria were as follows: (1) primary VUR with grades IV–V confirmed by voiding cystourethrography (VCUG) or contrast-enhanced voiding urosonography (ceVUS); (2) grade III VUR accompanied by breakthrough febrile upper urinary tract infection despite continuous antibiotic prophylaxis, and differential renal function (DRF) of the affected kidney < 40% or a significant decline in DRF on serial assessment (defined as an absolute reduction of ≥5% in ipsilateral DRF on follow-up DMSA compared with baseline measurement). A subset of patients underwent surgery primarily because of impaired DRF or progressive DRF decline, while the majority were operated on due to recurrent febrile UTI and high-grade reflux.

Patients were excluded if they had concomitant upper urinary tract anomalies (e.g., ureteral stricture, duplex collecting system) or secondary VUR due to underlying lower urinary tract pathology (e.g., posterior urethral valves or neurogenic bladder). Patients with ureteral dilation were included when tapering and reimplantation were feasible, and ureteral tapering was performed when indicated.

Among the included patients, 97 underwent the Politano–Leadbetter technique (Group P) and 82 underwent the Cohen technique (Group C). Baseline demographic and clinical characteristics are summarized in Table 1.

**Table 1:** Comparison of the characteristics of pediatric patients undergoing ureteral reimplantation between the Politano–Leadbetter and Cohen techniques.

Variables	Group P (Politano-Leadbetter technique)	Group C (Cohen technique)	*p*
No. of patients	97	82	
Sex			0.452
Male	59	44	
Female	38	38	
Laterality			0.807
Unilateral	67	53	
Bilateral	30	29	
Age (months), mean ± SD	60.12 ± 7.01	61.97 ± 9.25	0.102
Previous STING/Deflux injection	0	0	1.000
Ureteral diameter (mm), mean ± SD	11.77 ± 4.69	11.34 ± 4.86	0.557
VUR grade			0.796
III	12	14	
IV	64	52	
V	21	16	
History of febrile UTI	59	61	0.069
Breakthrough febrile UTI during CAP	5	8	0.576
Renal scarring on DMSA	43	49	0.054
DRF <40% or a significant decline in DRF on serial assessment	16	10	0.431

### Surgical techniques

The choice between Politano–Leadbetter and Cohen techniques was made by the attending surgeon based on ureteral anatomy, ureteral diameter, reflux laterality, feasibility of tunnel creation, surgeon experience, institutional practice evolution, and discussion with the guardians. The pneumovesicoscopic Cohen technique was introduced in our institution in 2019, whereas the pneumovesicoscopic Politano–Leadbetter technique was introduced in 2020.

Under general anesthesia, patients were placed in the lithotomy position. A 6-Fr or 8-Fr urethral catheter was inserted to completely drain the bladder. A cystoscope was introduced transurethrally and connected to a carbon dioxide (CO₂) insufflation system to maintain an intravesical pressure of 8–12 mmHg at a flow rate of 3–4 L/min. The bladder dome was identified externally, and two traction sutures were placed perpendicular to the patient's longitudinal axis to appose the bladder wall to the anterior abdominal wall. A 5-mm trocar was then inserted percutaneously and secured between the traction sutures, and a laparoscope was introduced to establish the CO₂ pneumovesicum working space. Two additional 3-mm trocars were placed bilaterally under endoscopic guidance as working ports.

### Cohen technique

The Cohen technique was performed as described by Yeung et al. (6) and Bi et al. (11). A circumferential incision was made around the affected ureteral orifice through the mucosal and muscular layers. The distal ureter was mobilized extravesically along the mesoureteral plane for 3–5 cm (adjusted according to ureteral diameter) and then retracted into the bladder through the original hiatus. A new mucosal orifice was created approximately 1–2cm superolateral to the contralateral ureteral orifice. A 3–5cm submucosal tunnel was developed between the original and new orifices. The ureter was advanced through the tunnel and anastomosed to the mucosa at the new orifice using interrupted absorbable sutures. The muscular and mucosal layers at the original hiatus were reapproximated and closed.

In cases of a dilated megaureter, excisional tapering was performed. After adequate distal mobilization into the pneumovesical space, the redundant antimesenteric ureteral wall was excised. Tapering was performed over an 8-Fr catheter template to standardize the lumen. The ureter was reconstructed using running or interrupted 5-0/6-0 absorbable sutures in a watertight manner, with careful preservation of the longitudinal adventitial blood supply. The tapered ureter was then repositioned into the newly created submucosal tunnel for reimplantation.

For bilateral Cohen procedures in Group C, two separate submucosal tunnels were created, and the ureters were cross-reimplanted to the contralateral superolateral position without using a common tunnel.

### Politano-Leadbetter technique

**Figure 1:**
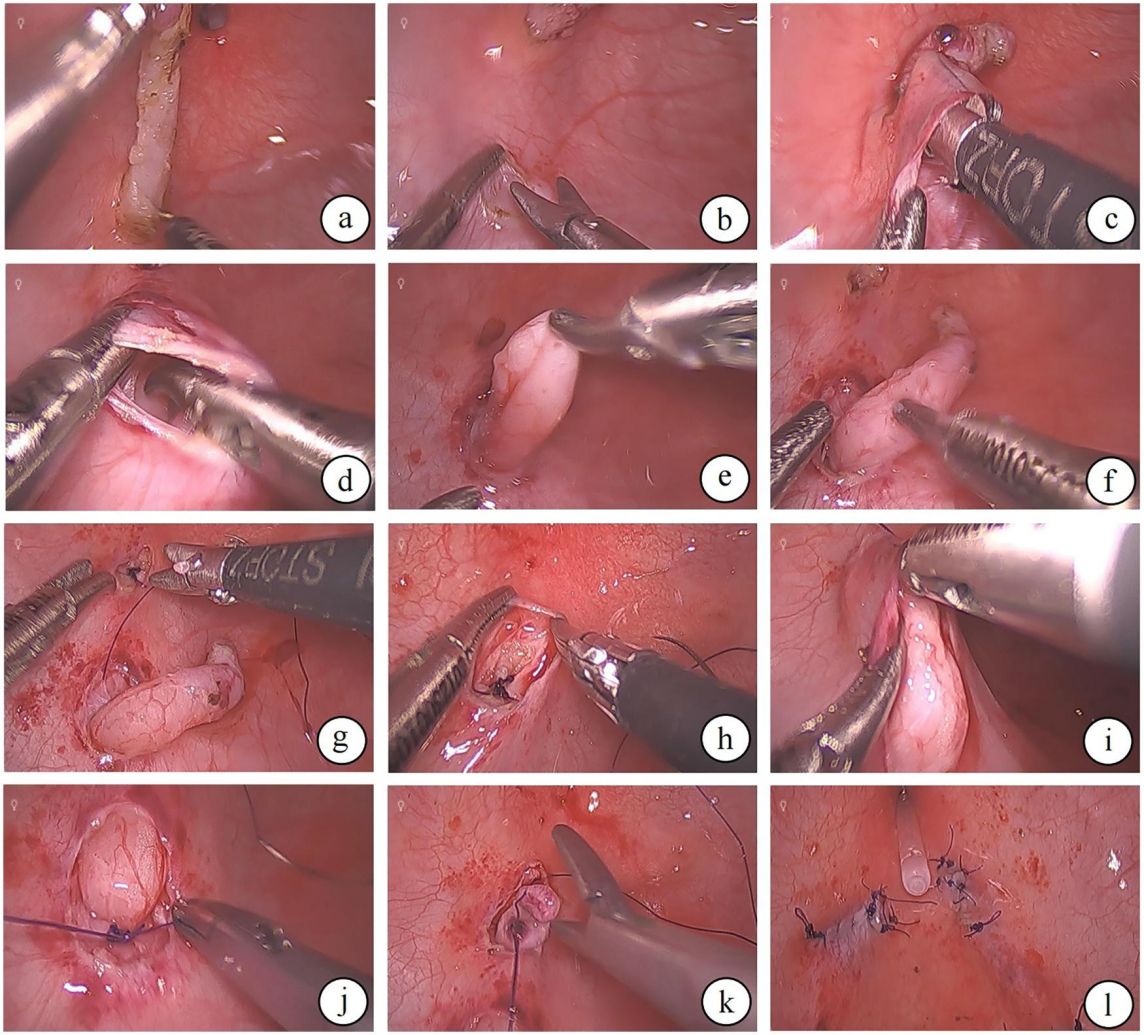
Surgical procedure of the Politano–Leadbetter technique for VUR. **(a)** The ureter is mobilized. **(b)** An incision is made in the bladder mucosa approximately 3 cm superior to the original ureteral orifice, following the projected course of the ureter. **(c)** A submucosal tunnel is created from the new hiatus toward the original orifice. **(d)** The detrusor muscle layer inferior to the new hiatus is dissected to access the extravesical space, with careful release of surrounding soft tissues to facilitate ureteral identification. **(e)** Under direct visualization, the ureter is drawn into the bladder through the new hiatus. **(f)** The ureter is checked to ensure the absence of kinking or torsion caused by adjacent tissue traction. **(g)** The detrusor muscle at the original orifice is reapproximated, and tunnel length is assessed. **(h)** If needed, the mucosal plane is further dissected from the original orifice toward the bladder neck to extend the tunnel length. **i** The ureter is passed through the submucosal tunnel. **(j)** The detrusor defect at the new hiatus was closed, with proximal fixation of the ureter. **(k)** The distal ureteral mucosa is anastomosed to the bladder mucosa with interrupted sutures. **(l)** Final appearance following bilateral ureteral fixation.

With a stable pneumovesicum, the Politano–Leadbetter procedure was performed in a manner similar to that described by Choi et al. (12). Distal ureteral mobilization was initiated in the same fashion as in the Cohen technique. After adequate length was obtained, gentle traction on the ureter was used to delineate its retrovesical course. An incision was made through the bladder mucosa and detrusor approximately 2–3 cm superior to the original ureteral orifice along the projected ureteral path to enter the extravesical space. The peritoneal reflection was carefully dissected to avoid injury to adjacent structures (e.g., vas deferens in males or fallopian tubes in females). The ureter was exteriorized through this new detrusor hiatus, and extravesical connective tissue was released to prevent kinking or torsion. A longitudinal submucosal tunnel was created from the new hiatus toward the original orifice, and the detrusor at the new hiatus was closed. When tapering was required, the technique was identical to that described for the Cohen procedure. The ureter was then repositioned through the tunnel and anastomosed to the original orifice with interrupted absorbable sutures. Any remaining mucosal defects were closed using continuous absorbable sutures (Figure 1). If the tunnel length was deemed insufficient, additional mucosal dissection toward the bladder neck was performed to extend the tunnel.

### Technical refinements for the politano–leadbetter procedure

**Figure 2:**
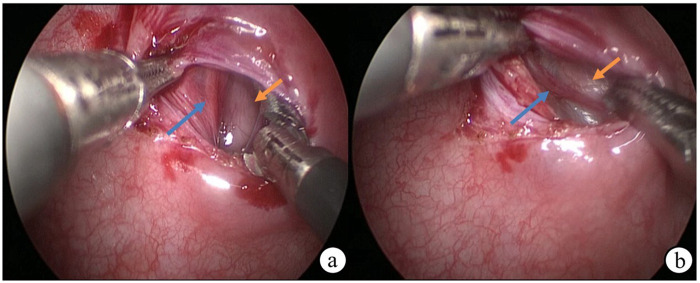
Intraoperative pneumovesicoscopic views during right-sided Politano-Leadbetter ureteral reimplantation, illustrating the critical anatomical relationship between the vas deferens and ureter. **(a)** View from the new hiatus, with the vas deferens (blue arrow) positioned medial to the ureter (yellow arrow). **(b)** The convergence point of the vas deferens and ureter is clearly visualized.

To simplify extravesical ureteral localization and mobilization during pneumovesicoscopic Politano–Leadbetter reimplantation, we used the following anatomical landmarks: (1) Vas deferens as a landmark: In male patients, the ureter converges with the vas deferens near the new hiatus on the posterior bladder wall, and the vas deferens then courses medially. Careful dissection between these structures facilitates ureter identification while protecting the vas deferens (Figure 2). (2) Peritoneal reflection: The peritoneal reflection serves as a critical boundary; maintaining peritoneal integrity helps prevent CO₂ leakage, pneumoperitoneum, and bladder collapse. When the reflection is positioned inferiorly, the ureter typically lies lateral to the peritoneum, providing a useful topographic guide. (3) Complete extravesical mobilization: Meticulous release of periureteral connective tissue is essential to ensure a tension-free course and reduce postoperative kinking or obstruction as the ureter is transposed through the new hiatus into the submucosal tunnel.

### Postoperative management

A double-J ureteral stent was routinely placed in the affected ureter at the end of the procedure. A 5-Fr stent was used, and the length was estimated according to the patient's age and height, generally calculated as age(years) + 10 cm. The stent was left *in situ* for 1–2 months and then removed cystoscopically. Postoperatively, a urethral catheter was routinely maintained for 3–5 days, and the timing of removal was determined based on urine output and urine appearance.

### Observation indicators and follow-up

The following variables were recorded and compared between the two groups: operative time, estimated blood loss, duration of urethral catheterization, length of hospital stay, and postoperative complications. Previous endoscopic subureteric injection, including STING or Deflux injection, was reviewed from the medical records. Follow-up was scheduled at 1, 3, and 6 months postoperatively and every 6 months thereafter. Considering the invasiveness and radiation exposure associated with routine VCUG, we did not perform VCUG in patients without clinical symptoms. VCUG was preferentially performed when clinically indicated (e.g., febrile UTI, flank pain or suspected recurrence), whereas ceVUS was used as an alternative modality during follow-up for patients with mild clinical symptoms (e.g., lower urinary tract symptoms). All patients who developed febrile urinary tract infection during follow-up underwent repeat VCUG to rule out recurrent VUR.

Clinical success was defined as the absence of recurrent febrile UTI, absence of symptoms suggestive of persistent reflux or anastomotic obstruction, and no need for secondary anti-reflux surgery during follow-up. Imaging-confirmed anatomical success was defined as complete resolution of VUR or downgrading to grade I on postoperative VCUG or ceVUS without evidence of anastomotic obstruction. Asymptomatic patients without postoperative imaging were considered to have achieved clinical success but were not included in the calculation of imaging-confirmed anatomical success.

### Statistical analysis

Statistical analyses were performed via SPSS software (version 22.0). Continuous variables were tested for normality using the Shapiro–Wilk test. Normally distributed variables are presented as mean ± standard deviation and were compared using Student's unpaired t-test. Non-normally distributed variables are presented as median and interquartile range and were compared using the Mann–Whitney U test. Categorical variables are presented as numbers and percentages and were compared using the chi-square test or Fisher's exact test, as appropriate. A two-sided p value <0.05 was considered statistically significant.

## Results

All 179 patients successfully underwent the pneumovesicoscopic ureteral reimplantation procedure without conversion to open surgery. For patients with unilateral VUR, the operative time was 119.85 ± 29.13 minutes in Group P and 116.21 ± 22.04 minutes in Group C (*p* = 0.437). The estimated blood loss was 6.15 ± 0.98 mL in Group P and 6.02 ± 0.91 mL in Group C (*p* = 0.454). The duration of urethral catheterization postoperatively was 4.31 ± 0.59 days in Group P and 4.53 ± 0.85 days in Group C (*p* = 0.112). The hospitalization duration was 6.34 ± 1.71 days for Group P and 6.42 ± 0.93 days for Group C (*p* = 0.745). The incidence of complications was 7.5% in Group P and 15.1% in Group C (*p* = 0.217) (Table 2).

**Table 2:** Comparisons of the peri- and postoperative parameters and the Politano–Leadbetter and Cohen techniques in unilateral patients.

Variables	Group P (Politano-Leadbetter technique)	Group C (Cohen technique)	*p*
No. of patients	67	53	
operative time (minutes)	119.85 ± 29.13	116.21 ± 22.04	0.437
estimated blood loss (mL)	6.15 ± 0.98	6.02 ± 0.91	0.454
duration of urethral catheterization (days)	4.31 ± 0.59	4.53 ± 0.85	0.112
hospitalization (days)	6.34 ± 1.71	6.42 ± 0.93	0.745
complications (case, %)	5 (7.5%)	8 (15.1%)	0.217
hemorrhage	1	0	
urinary extravasation	2	5	
febrile urinary tract infections	0	1	
anastomotic obstruction	0	1	
recurrent reflux	1	0	
hydronephrosis	1	1	

For patients with bilateral VUR, the operative time was 155.00 ± 48.31 minutes in Group P and 137.24 ± 38.07 minutes in Group C (*p* = 0.102). The estimated blood loss was 7.33 ± 1.27 mL in Group P and 7.24 ± 1.48 mL in Group C (*p* = 0.803). The duration of urethral catheterization was 5.07 ± 2.16 days in Group P and 4.82 ± 1.71 days in Group C (*p* = 0.623). The hospitalization duration was 7.10 ± 1.74 days for Group P and 6.79 ± 0.94 days for Group C (*p* = 0.395). The incidence of complications was 13.3% in Group P and 17.2% in Group C (*p* = 0.677) (Table 3).

**Table 3:** Comparisons of the peri- and postoperative parameters and the Politano–Leadbetter and Cohen techniques in bilateral patients.

Variables	Group P (Politano-Leadbetter technique)	Group C (Cohen technique)	*p*
No. of patients	30	29	
operative time (minutes)	155.00 ± 48.31	137.24 ± 38.07	0.102
estimated blood loss (mL)	7.33 ± 1.27	7.24 ± 1.48	0.803
duration of urethral catheterization (days)	5.07 ± 2.16	4.82 ± 1.71	0.623
hospitalization (days)	7.10 ± 1.74	6.79 ± 0.94	0.395
complications (ureter, %)	4 (13.3%)	5 (17.2%)	0.677
hemorrhage	1	0	
urinary extravasation	0	3	
recurrent febrile urinary tract infections	1	0	
anastomotic obstruction	1	1	
recurrent reflux	0	1	
hydronephrosis	1	0	

**Figure 3:**
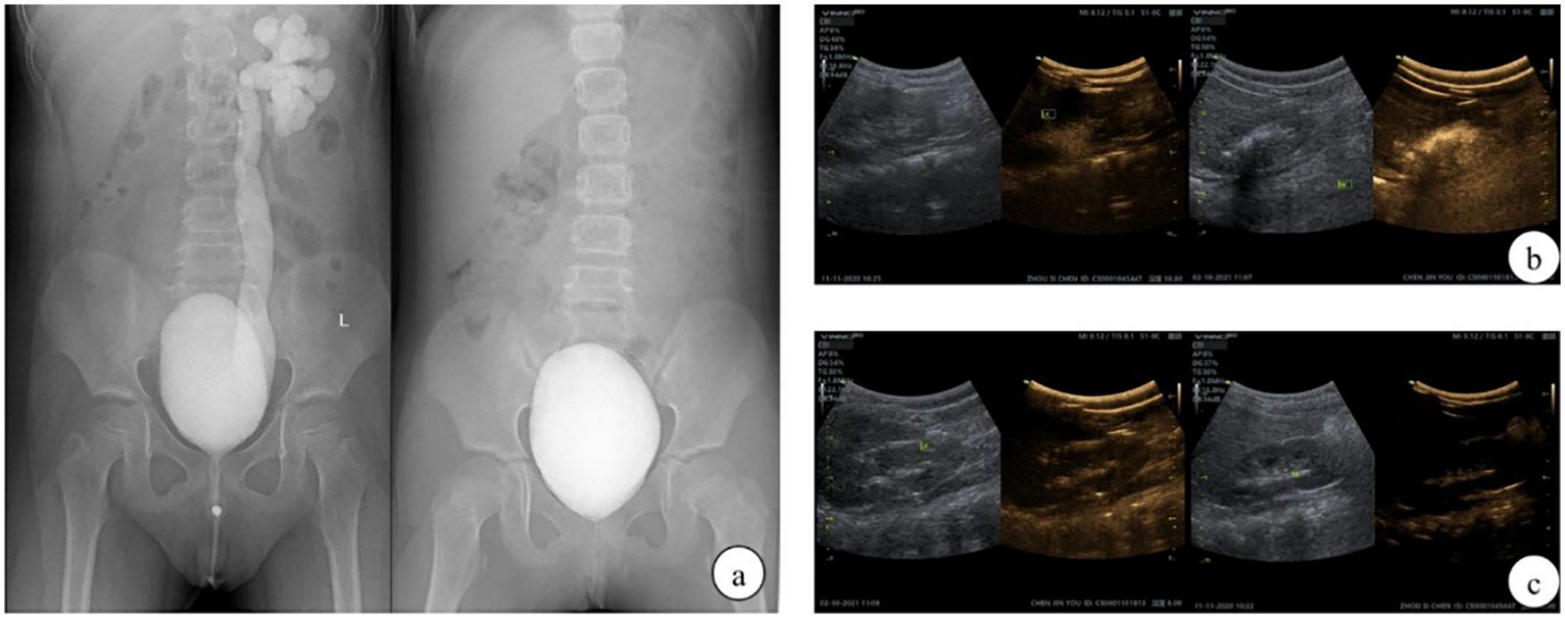
Postoperative follow-up findings. **(a)**Preoperative VCUG revealed left-sided grade IV-V VUR; no evidence of reflux was observed in the 6-month postoperative follow-up study. **(b)** Preoperative ceVUS revealed bilateral grade IV VUR. **(c)** Follow-up ceVUS at 6 months after surgery showing no significant reflux.

Overall, there were no statistically significant differences between the two techniques in perioperative outcomes for either unilateral or bilateral VUR. All children achieved unobstructed voiding after catheter removal. Follow-up examinations using VCUG or ceVUS demonstrated improvement in ureteral dilation compared with preoperative status (Figure 3). In accordance with the study protocol, postoperative VCUG was performed in 25 patients, ceVUS in 110 patients, while 44 patients remained entirely asymptomatic and consequently did not undergo any postoperative invasive investigations. Surgical failure occurred in 2 patients (2 ureters) in Group P and 3 patients (3 ureters) in Group C; therefore, the overall clinical success rate per-ureter basis was 98.4% in Group P and 97.3% in Group C, while the imaging-confirmed anatomical success rate per-ureter basis was 98.1% in Group P and 96.3% in Group C. Complication rates are reported per patient because events such as hemorrhage, urinary extravasation, and febrile urinary tract infection cannot be reliably assigned to an individual ureter in bilateral cases. When analyzed per patient, there were no significant differences between groups (Tables 2, 3).

## Discussion

Over the past decade, the management of pediatric primary VUR has progressively shifted toward minimally invasive approaches. Endoscopic injection of bulking agents is generally considered suitable for low-grade VUR; however, concerns remain regarding durability beyond five years and the potential need for repeat injections or the risk of ureteral obstruction (13). The extravesical Lich–Gregoir technique, including robotic-assisted variants, yields favorable outcomes but may be associated with pelvic nerve injury, postoperative voiding dysfunction, and interference with adjacent abdominopelvic structures (14). In contrast, pneumovesicoscopic intravesical ureteral reimplantation—most commonly performed using the Cohen or Politano–Leadbetter techniques—avoids entry into the peritoneal cavity, minimizes surgical trauma, and enables reconstruction of the ureterovesical junction in a near-physiological manner (1, 10, 15, 16). These characteristics make transvesicoscopic approaches an appealing option for pediatric VUR repair.

In our institution, the pneumovesicoscopic Cohen technique was adopted in 2019, followed by implementation of the Politano–Leadbetter procedure in 2020. In the present series, the Politano–Leadbetter group demonstrated a longer mean operative time, which is consistent with the greater technical complexity of this approach. In particular, identification and mobilization of the ureter through the newly created detrusor hiatus can be challenging and requires refined laparoscopic skills and familiarity with extravesical anatomy. As outlined in the Materials and Methods, we found that standardized anatomic orientation and consistent operative steps facilitated this phase and helped mitigate the learning curve for the Politano–Leadbetter procedure.

Pneumovesicoscopic ureteral reimplantation has procedural characteristics that may reduce certain difficulties encountered in the traditional open Politano–Leadbetter operation. The transvesicoscopic view provides direct visualization through the new hiatus, which can aid anatomic identification and improve operative precision. In our experience, applying stable anatomic landmarks (described in detail in the Methods) improved confidence during extravesical dissection and may reduce the risk of injury to adjacent structures. Moreover, complete ureteral mobilization and a tension-free course are essential to preserve a physiological ureteral trajectory. This consideration is clinically relevant because the Politano–Leadbetter technique maintains a more anatomical ureteral path and preserves the possibility of future retrograde endoscopic access, whereas the cross-trigonal Cohen reimplantation alters the intravesical ureteral course.

Postoperative outcomes and complications remain central to assessing the efficacy of these approaches. In this cohort, complications included hemorrhage, urinary extravasation, febrile urinary tract infection (UTI), anastomotic obstruction, and recurrent reflux. Importantly, surgical failure was observed in both groups. A review of operative records and surgical videos suggested that possible technical contributors to surgical failure may include anastomotic tension, inadequate ureteral mobilization, ischemic change, excessive tapering, or insufficient submucosal tunnel length. However, because only five failures occurred, these observations should be interpreted as hypothesis-generating rather than definitive causal conclusions. Urinary extravasation typically occurred early after surgery, likely due to transient edema or temporary stent blockage, and resolved with conservative management in nearly all cases; only one patient required stent revision. Collectively, these findings highlight that, despite excellent short-term outcomes, meticulous attention to tension-free anastomosis, preservation of ureteral blood supply, and appropriate ureteral tailoring remains critical for both procedures.

From a technical and anatomical standpoint, the Politano–Leadbetter technique may offer several potential advantages. First, the more proximally positioned new hiatus can facilitate additional ureteral mobilization, which may improve exposure for tailoring or resection of pathological segments in tortuous or markedly dilated ureters and may reduce the risk of postoperative kinking. Second, by maintaining a near-physiological ureteral position and preserving the original orifice location, the Politano–Leadbetter technique may better preserve the feasibility of future retrograde endoscopic interventions compared with cross-trigonal reimplantation. Third, creation of the submucosal tunnel may be more straightforward in some cases because the tunnel direction can align more naturally with laparoscopic instrument trajectories, and the hiatus position can be adjusted to optimize the tunnel length-to-diameter ratio. In contrast, the Cohen technique requires more extensive trigonal dissection, where tissue density and mucosal adherence may increase the risk of perforation and may limit tunnel length in substantially dilated ureters. These considerations suggest that the Politano–Leadbetter approach may be particularly useful in anatomically complex cases; nevertheless, careful patient selection and surgical expertise remain essential.

This study has several limitations. This was a retrospective single-center study, and the choice of surgical technique was not randomized; therefore, selection bias cannot be excluded. The allocation of PL or Cohen reimplantation was influenced by surgeon preference, ureteral anatomy, and institutional practice evolution. Incomplete postoperative imaging follow-up represents an important limitation of this study, as asymptomatic persistent reflux could not be fully excluded in patients without postoperative VCUG or ceVUS. This may have introduced selection bias and potentially overestimated the true anatomical success rate. However, in an additional sensitivity analysis restricted to ureteral units with confirmed postoperative imaging, the success rates remained similar to those observed in the overall ureteral-unit cohort. In the PL group, the overall clinical success rate per-ureter basis was 98.4% (125/127), compared with 98.1% (102/104) in the imaging-confirmed anatomical success subgroup. In the Cohen group, the corresponding rates were 97.5% (117/120) and 96.3% (77/80), respectively. These findings suggest that incomplete postoperative imaging follow-up was unlikely to substantially change the main efficacy conclusions, although the possibility of undetected asymptomatic reflux cannot be completely excluded. Both VCUG and ceVUS were used during follow-up, which may have introduced heterogeneity in radiological outcome assessment. Postoperative renal functional imaging was not uniformly performed in all patients, limiting evaluation of long-term renal functional preservation. The number of surgical failures was small, precluding robust analysis of predictors or mechanisms of failure. Finally, because both procedures were adopted relatively recently, a residual learning-curve effect may have influenced operative outcomes.

In conclusion, pneumovesicoscopic ureteral reimplantation is a safe and effective minimally invasive option for pediatric VUR in the short term, with minimal trauma and rapid recovery. Both the Politano–Leadbetter and Cohen techniques achieved high success rates. While the Politano–Leadbetter procedure may be technically more demanding, it offers a more physiological ureteral course and flexible tunnel construction, which may be advantageous in selected patients.

## Data Availability

The original contributions presented in the study are included in the article/Supplementary Material, further inquiries can be directed to the corresponding author.
