# Supplementary material for: Pneumovesicoscopic ureteral reimplantation for pediatric vesicoureteral reflux: Politano-Leadbetter vs. Cohen technique
Source: Front Pediatr. 2026 Jul 21;14:1855283. doi: 10.3389/fped.2026.1855283 (PMC13433845; doi:10.3389/fped.2026.1855283)
Supplement: Supplementary file 1 [file Table1.docx]

**Supplementary table 1** Composition of High-risk Grade III VUR Patients in Group P and Group C

| Variables | Group P (Politano-Leadbetter technique) | Group C (Cohen technique) | *p* |
| --- | --- | --- | --- |
| No. of patients | 12 | 14 |  |
| Sex |  |  | 0.652 |
| Male | 10 | 10 |  |
| Female | 2 | 4 |  |
| Laterality |  |  | 1.000 |
| Unilateral | 12 | 14 |  |
| Bilateral | 0 | 0 |  |
| Age (months), mean±SD | 63.51 ± 5.05 | 62.33 ± 8.19 | 0.672 |
| Previous STING/Deflux injection | 0 | 0 | 1.000 |
| Ureteral diameter (mm), mean±SD | 11.01 ± 2.19 | 10.83 ± 3.37 | 0.876 |
| Breakthrough febrile UTI during CAP | 9 | 12 | 0.631 |
| DRF <40% or a significant decline in DRF on serial assessment | 7 | 9 | 0.703 |

**Supplementary table 2** chronological distribution of cases

| Period | Group P (Politano-Leadbetter technique) | Group C (Cohen technique) |
| --- | --- | --- |
| 2021 | 14 | 31 |
| 2022 | 20 | 17 |
| 2023 | 38 | 23 |
| 2024 | 25 | 11 |
